# Automated ribotyping and antibiotic resistance determining of *Bacillus* spp from conjunctiva of diabetic patients

**Published:** 2014-02

**Authors:** Sertaç Argun Kıvanç, Merih Kıvanç, Gülay Güllülü

**Affiliations:** 1 Erzurum Region Education and Research Hospital, Department of Ophthalmology, Erzurum, Turkey; 2 Anadolu University, Faculty of Science, Department of Biology, Eskisehir Turkey; 3 Armedica Eye Hospital, Kocaeli, Turkey

**Keywords:** Antibiotic resistance, Automated ribotyping, * Bacillus* spp., Diabetic eyes

## Abstract

***Objective(s):*** We aimed to characterize the phenotype and genotype of *Bacillus *spp isolated from diabetic patients’ eyes, by studying the drug sensitivity patterns with a disc-diffusion method.

***Materials and Methods:*** Fifty eyes of 25 patients with type II diabetes mellitus, with at least 10 years of diabetes history, were included in the study. We analyzed the eyes for the presence of *Bacillus* spp.; presumptive isolates were identified by morphological, and biochemical tests, and confirmed by the VITEK system. Automated *Eco*RI ribotyping was performed with a RiboPrinter^®^ Microbial Characterization System. We determined the antibiotic resistance of the isolates by the Kirby–Bauer disc diffusion test.

***Results:*** Seven out of 25 patients were on insulin treatment; 7 on oral anti-diabetic medication; and 11 on combination therapy of insulin and oral medications. Among the 28 *Bacillus* spp isolates, 14 were *B. cereus*, 11 were *B. pumilus, *2 were *B.* *mojavensis *and 1 was *B. subtilis.* Almost all the strains were either resistant or multiresistant, particularly towards cefuroxime, methicillin, and ceftazidime.

***Conclusion: ***Diabetic patients seem to be more prone to *B. cereus* infections than healthy individuals. It would be greatly beneficial to understand and recognize the prevalence of microorganisms and their resistance patterns for better outcome in ocular surgeries.

## Introduction

*Bacillus* spp and coagulase-negative staphylococci are the most common causes of Gram-positive endogenous endophthalmitis (1). Bacteria of the genus *Bacillus *are ubiquitous in the natural environment. In the world of clinical microbiology, *Bacillus* spp have potentially important roles as “contaminants” as well as pathogens (2). The genus *Bacillus *comprises a very large and diverse group, the members of which are either aerobes or facultative anaerobes. *Bacillus* spp are Gram-positive rods that are capable of forming endospores; they usually produce catalase and tolerate extremes of temperature and moisture (3). Their phylogenetic taxonomy places them in the phylum *Firmicutes*, class *Bacilli*, order *Bacillales*, and family *Bacillaceae*. In the *Bacillaceae* family, no other related spp are as important as the *Bacillus* spp The order *Bacillales *includes most of the familiar Gram-positive human pathogens. *Bacillus cereus *is one of the most common causes of post-traumatic and post-operative endophthalmitis, particularly in the presence of retained intraocular foreign bodies (4).* Bacillus *spp is a major cause of rapid blinding in post-traumatic and endogenous endophthalmitis cases. The majority of patients with *Bacillus* endophthalmitis lose significant visual function or the eye itself in less than few days (5-9). *B. pumilus, B. licheniformis,* and *B. subtilis* are among the species comprising the *B. subtilis* group of aerobic spore-forming organisms, all of which are very similar (10). Despite intensive medical and surgical intervention, patients typically retain poor vision (1). 

Compromised immunity is an important factor in the development of endogenous endophthalmitis. In a review, 56% of patients with endogenous bacterial endophthalmitis were also immunocompromised, and diabetes was the most common underlying disease involved (1). The increased risk of infection in diabetics has been well documented; however, no correlation has been shown between diabetes and post-operative or post-traumatic endophthalmitis. Links between underlying ocular diseases associated with diabetes (e.g., diabetic retinopathy) have not been established. *B. cereus* is by far the most common cause of *Bacillus* endophthalmitis. However, endophthalmitis can also becaused by *B. thuringiensis*, a bacterium that is commonly used for organic gardening and farming.

*Bacillus thuringiensis* is genetically and phenotypically similar to *B. cereus *(11, 12). For *B. cereus* and *B. thuringiensis*, the quorum-sensing transcriptional regulator—plcR—controls the expression of many extracellular virulence factors (13). Wild-type *Bacillus* causes severe intraocular inflammation in 12 hr. The ability of *B. cereus* toxins to induce the type of damage seen in endophthalmitis was shown in a mouse model of sterile endophthalmitis, in which bacterial supernatants from wild type and plcR-deficient *B. cereus* were examined. Supernatant from the wild-type *B. cereus* caused rapid loss of retinal function and more severe inflammation than did the supernatant from plcR-deficient *B. cereus *(14). In terms of individual toxins, those tested to date (hemolysin BL, phosphatidylinositol-specific phospholipase C, and phosphatidylcholine-specific phospholipase C) contributed little to the overall pathogenesis of experimental *B. cereus* endophthal -mitis (15, 16). Taken together, these data highlight the importance of quorum-sensing to the pathogenicity of *Bacillus* endophthalmitis (6-9).

*B. pumilus *is highly resistant to extreme environmental conditions such as low or no nutrient availability, desiccation, irradiation, H_2_O_2_, and chemical disinfections (17). *B. cereus, B. licheniformis*, and *B.* *pumilus* may be more pathogenic in immunosuppressed hosts than other common *Bacillus *species (*B. subtilis* or *B. megaterium*) (18). However, *B. pumilus* has been rarely reported as a human pathogen (19). *B. pumilus* has toxic properties; it has cytopathic effects in Vero cells, hemolytic activity, the capacity for lecithinase production, and proteolytic action on casein (20). Little information has been published about *B. mojavensis* (21).

The present study has focused on the phenotype and genotype characterization of *Bacillus *spp obtained from the healthy conjunctiva of eyes of diabetic patients on the basis of their drug-sensitivity patterns assessed by the disc diffusion method. 

## Materials and Methods

***Subject***

A total of 25 patients, (15 women [60%] and 10 men [40%]; mean age: 59.54 ± 6.72 years) with Type II diabetes mellitus (n = 50 eyes) , with at least 10 years of diabetes history, and who had visited our ophthalmology department for routine diabetic control, without ocular infection or ocular allergic symptoms, were included in this study. Microbiologic sampling from conjunctival fornices was performed twice for both eyes with sterile cotton swabs, without topical anesthetic drops. Using sterile Stuart’s swabs, we obtained swabbed samples from the conjunctiva of each patient, which were then placed in Stuart’s transport medium and transferred to the microbiology laboratory. Informed consent was obtained from all the patients prior to conjunctival sampling.

***Isolation of Bacillus spp***

The conjunctival swabs were streaked on culture media. The following culture media: blood agar (5% sheep) and blood and mannitol egg-yolk-polymyxin agar (MYP-agar) were used. The culture media were incubated at 37°C to permit bacterial growth; the cultures were retained for 3 days to ascertain bacterial growth. 

***Bacterial identification***

Colonies representing the most number of bacteria in each sample were subcultured in MYP-agar and blood agar by streaking on the same fresh medium and incubating at 37^o^C for 24–48 hr. The isolates were screened by colonial morphology, Gram staining, and spore formation. Further, we investigated the following parameters: coagulase activity, hemolysis, oxidation/fermentation of glucose, motility, catalase, oxidase, coagulase, growth in anaerobic medium, starch hydrolysis, casein hydrolysis, and gelatin hydrolysis (22). In addition, Voges–Proskauer and indol tests were done and nitrate reduction, starch hydrolysis, and mannitol utilization were also evaluated (23). The strains were further identified using the VITEK system (bioMerieux), according to the manufacturer instructions. The VITEK identification system is also a carbon-source utilization test. The reliability of these systems depends upon the number and diversity of bacteria in the databases.

Automated *Eco*RI ribotyping was performed with a RiboPrinter^®^ Microbial Characterization System (Dupont Qualicon). The standard *Eco*RI DNA preparation kit was used based on the manufacturer protocol. Pure culture samples were obtained from agar plates incubated for 24–48 hr at 30°C. The microbial samples were subsequently analyzed according to the manufacturer instructions. The ribotype profiles of the isolates were compared with the reference DuPont identification database DUP2003. The identification of each isolate was done when the corresponding pattern matched one of the patterns of the DuPont Identification Library with a similarity of ≥0.85. The isolates were automatically grouped in ribogroups by the RiboPrinter^®^ on the basis of the similarity of the respective ribotype patterns. 

The generated Riboprinter^®^ patterns were analyzed with the Finger Printing II software (DuPont Qualicon ,USA), and a dendrogram was generated by using the Unweighted Pair Group Method using arithmetic Averages (UPGMA) and Pearson correlation coefficients (optimization, 1.56%). 

**Table 1:** *Eco*RI ribotyping profiles of the *Bacillus* spp isolated from conjunctiva of diabetic patients

No	Sample no	Dupont ID label	Ribogroup	DUP number	Similarity
I	KA 17.7-1	*B. cereus*	*Eco*RI 425-114-S-4	DUP-6066	0.85
	PCA 15.4-1	*B. cereus*	*Eco*RI 425-114-S-4	DUP-6066	0.84
	PCA 15.3	*B. cereus*	*Eco*RI 425-114-S-4	DUP-6066	0.87
	PCA 17.2	*B. cereus*	*Eco*RI 425-114-S-4	DUP-6066	0.85
	PCA 15.5-2	*B. cereus*	*Eco*RI 425-114-S-4	DUP-6066	0.85
	KA17.11	*B. cereus*	*Eco*RI 425-114-S-4	DUP-6066	0.87
	PCA 17.10	*B. cereus*	*Eco*RI 425-114-S-4	DUP-6066	0.85
	KA17.8	*B. cereus*	*Eco*RI 425-114-S-4	DUP-6066	0.87
II	KA 50.1	*B. cereus*	*Eco*RI 425-126-S-5	DUP-6066	0.83
III	KA 22.3	*B. cereus*	*Eco*RI 425-114-S-4	DUP-13209	0.86
	KA 17.4	*B. cereus*	*Eco*RI 425-114-S-4	DUP-13209	0.84
IV	KA 17.6-1	*B. cereus*	E*co*RI 425-126-S-8	DUP-13209	0.84
	KA 52	*B. cereus*	*Eco*RI 425-126-S-8	DUP-6092	0.87
V	KA 49.2	*B. cereus*	*Eco*RI 425-114-S-8	DUP-6092	0.85
VI	PCA 36.1	*B. pumilus*	*Eco*RI 425-122-S-2	DUP-6073	0.90
	KA 17.5.1	*B. pumilus*	*Eco*RI 425-122-S-2	DUP-6073	0.93
	KA 17.5.2	*B. pumilus*	*Eco*RI 425-122-S-2	DUP-6073	0.92
	PCA 39.3	*B. pumilus*	*Eco*RI 425-122-S-2	DUP-6073	0.84
	KA 35.1	*B. pumilus*	*Eco*I 425-122-S-2	DUP-6073	0.92
VII	PCA 4.2	*B. pumilus*	*Eco*RI 425-129-S-4	DUP-11052	0.93
	PCA 9.4	*B. pumilus*	*Eco*RI 425-129-S-4	DUP-11052	0.92
VIII	PCA 36.2	*B. pumilus*	*Eco*RI 425-122-S-2	DUP-11055	0.87
	PCA 35.1	*B. pumilus*	*Eco*RI 425-122-S-2	DUP-11055	0.78
	PCA 31	*B. pumilus*	*Eco*RI 425-122-S-2	DUP-11055	0.81
	KA 40	*B. pumilus*	*Eco*RI 425-122-S-2	DUP-11055	0.86
IX	PCA 24.1	*B. mojevensis*	*Eco*RI 425-114-S-2	DUP-13240	0.86
	PCA 34.1	*B. mojevensis*	*Eco*RI 425-114-S-2	DUP-13240	0.86
X	PCA 11.2	*B. subtilis*	*Eco*RI 425-129-S-1	DUP-18130	0.94

All strains were stocked in 10% glycerol and stored at -80°C. Working cultures were stored at 5°C and periodically transferred.

***Antibiotic sensitivity testing***

The antimicrobial resistance patterns of isolates were determined using the agar disc-diffusion method. Bacteria were suspended in sterile 0.85% saline to a turbidity matching that of a McFarland No. 2 standard (bioMe´rieux, Marcy l’Etoile, France), diluted 1:20, and streaked on Mueller–Hinton agar. Discs containing the following antibacterial agents were used: gatifloxacin (5 µg), cefuroxime (30 µg), ceftazidime (30 µg), vancomycin (30 µg), gentamicin (10 µg), amikacin (30 µg), ciprofloxacin (5 µg), lomefloxacin (10 µg), moxifloxacin (5 µg), and methicillin (10 U). Plates were incubated at 37°C for 24–48 hr depending on the organisms contained therein. The strains were characterized as sensitive, intermediate, or resistant based on the size of the inhibition zones around each disc, according to the National Committee for Clinical Laboratory Standards (CLSI) criteria (24).

## Results

Among 25 patients,15 women (60%) and 10 men (40%), (mean age: 59.54 ± 6.72 years), 7 were on insulin treatment, 7 were using oral anti-diabetics, and 11 were using a combination of insulin and oral anti-diabetics. The mean HbA1c level was 8.3 ± 1.61. In addition to diabetes mellitus, 17 patients had hypertension, 12 had hypercholesterolemia, 2 had coronary artery disease, and 1 had breast cancer.

**Figure 1:**
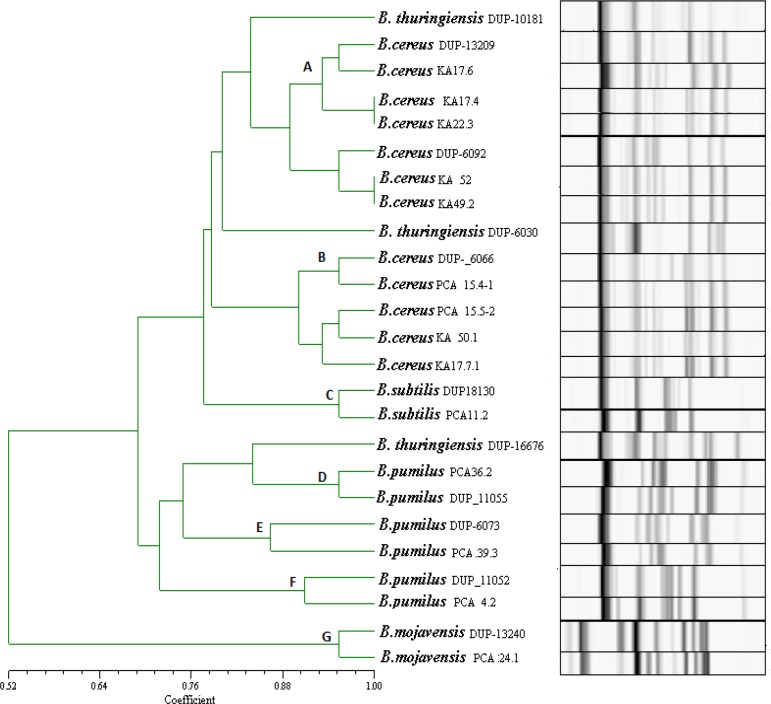
Cluster analysis of the *Bacillus *spp isolates from conjunctiva. Dendrogram based on UPGMA cluster analysis

No growth was observed in 14 of 25 patients (56%). *Bacillus *spp were isolated from 15 of 50 eyes (isolates obtained from one eye in 7 patients, and both eyes in 4 patients); 5 of these isolates were isolated from men and 10 from women. In the present study, 28 isolates were recovered from diabetic patients and identified using phenotypic and genotypic tests. The *Bacillus* spp isolates were initially identified based on their colonial morphology on plates, cellular appearance as viewed by light microscopy, and production of ovoid terminal or subterminal spores. On the basis of the results of the carbohydrate fermentation reactions and physiological and morphological tests, these samples were identified as *B. cereus, B. pumilus, B. mojevensis, *and* B. subtilis. *The predominant microbial flora was* B. cereus *followed by* B. pumilus.* The results obtained by *Eco*RI ribotyping confirmed the presumptive classification of the isolates within the species as the *Bacillus* spp Based on the preset identification similarity threshold of 0.86; all the strains were automatically identified using the RiboPrinter^®^. *B. cereus, B. pumilus, B. mojevensis,* and* B. subtilis* were detected using the RiboPrinter^®^ system. *Eco*RI ribotyping differentiated the isolates into 8 distinct ribotypes (Table 1). The similarity among these 8 ribogroups ranged from 0.78 to 0.97. The ribogroups belonged to 7 different DUP-IDs. These data show the high similarity inherent to the *Bacillus* strains isolated from the conjunctivas of patients with diabetes mellitus. *B. cereus* was the most common bacteria isolated, accounting for 14 out of the 28 isolates (50%). The other *Bacillus* spp isolated, included *B. pumilus* (11 isolates), *B. subtilis* (1 isolate)*, *and *B. mojevensis *(2 isolates)*.* The RiboPrinter^®^ results are shown in Figure 1. The 7 DUP-IDs were allocated to the evolutionary lineages known for *Bacillus* spp In particular, DUP-IDs 13209 and DUP-IDs 6092 were classified as Lineage I; DUP-IDs 6066, as Lineage II; and DUPIDs 18130, as Lineage III; DUP-IDs 11055, as Lineage IV; DUP-IDs 6073, as Lineage V; DUP-IDs 11052, as Lineage VI; and DUP-IDs 13240, as Lineage VII (Table 1). No atypical profile or profiles belonging to all lineages were found. The dendrogram patterns generated from ribotype data produced 7 clusters (Figure 1). The threshold regarding the measure of similarity was fixed at 0.85%. Cluster A consisted of 5 isolates; Cluster B, 9 isolates; Cluster C, 1 isolate; Cluster D, 4 isolates; Cluster E, 5 isolates; and Clusters F and G consisted of 2 isolates each from diabetes patients. The results of the cluster analysis allowed us to confirm the existence of a widespread population of *Bacillus *spp characterized by the diffusion of highly similar strains in diabetes patients.

**Table 2:** Susceptibility prevalence for *Bacillus spp * isolates (mm)

Strain	Ribogrup	Cefuroxime	Methicillin	Ceftazidime	Ciprofloxacin	Gentamicin	Amikacin	Vancomycin	Gatifloxacin	Lomefloxacin	Moxifloxacin
*B. cereus* KA 17.4	EcoRI-114-S-4	R	R	R	30	26	23	20	29	29	31
*B.cereus* KA 17.8	EcoRI-114-S-4	R	R	R	22	28	23	17	29	27	27
*B. cereus* PCA 17.2	EcoRI-114-S-4	11	R	13	27	26	23	19	28	33	32
*B.cereus* KA 17.11	EcoRI-114-S-4	7	R	13	40	36	25	20	30	33	34
*B. cereus* PCA 15.3	EcoRI-114-S-4	13	R	11	37	22	24	21	33	33	31
*B. cereus* KA 17.10	EcoRI-114-S-4	13	R	13	33	34	32	22	39	32	37
*B. cereus* PCA 15.5-2	EcoRI-114-S-4	R	R	10	36	29	41	24	37	24	43
B. cereus KA17.7-1	EcoRI-114-S-4	R	R	12	35	22	24	20	30	32	32
*B. cereus* KA 49.2	EcoRI-114-S-8	R	R	10	17	32	41	20	25	30	17
B. cereus KA 22.3	EcoRI-114-S-4	11	12	R	36	35	33	24	38	35	37
*B.cereus* PCA 15.4-1	EcoRI-114-S-4	12	R	R	50	25	41	21	28	27	40
*B. cereus* PCA 7.2	EcoRI-114-S-4	11	R	R	30	22	20	19	37	40	32
*B.cereus* KA 52 H(+)	EcoRI-114-S-8	R	10	22	34	16	22	20	36	15	28
*B. cereus* KA 17.6-1	EcoRI-126-S-8	39	15	31	R	R	28	48	51	R	R
*B. pumilus* PCA 35.1	EcoRI-122-S-2	12	9	R	30	31	29	21	40	33	43
*B.pumilus* KA 40	EcoRI-122-S-2	13	21	28	34	21	39	42	43	30	45
*B. pumilus *PCA 31	EcoRI-122-S-2	15	13	R	30	25	22	16	35	27	43
*B. pumilus *PCA 36.1	EcoRI-122-S-2	42	14	24	32	58	34	49	38	36	40
*B. pumilus *PCA 36.2	EcoRI-122-S-2	40	15	25	32	56	35	48	38	37	39
*B. pumilus* KA 17.5 -2	EcoRI-122-S-2	R	R	R	29	22	32	23	35	30	33
*B. pumilus* KA 17.5 -1	EcoRI-122-S-2	R	10	R	29	23	33	23	36	31	32
*B. pumilus* PCA 39.3	EcoRI-122-S-2	12	R	R	34	22	22	21	34	32	33
*B. pumilus* PCA 4.2	EcoRI-129-S-4	25	R	R	27	25	31	36	18	37	40
*B. pumilus* PCA 9.4	EcoRI-129-S-4	14	R	R	36	20	26	27	40	33	38
*B.mojevensis PCA 34.1*	EcoRI-114-S-2	30	15	15	25	39	55	31	35	R	37
*B.mojevensis* PCA 24.1	EcoRI-114-S-2	R	R	10	31	50	26	19	31	40	38
*B. subtilis* PCA 11.2- 1	EcoRI-129-S-1	36	19	28	35	24	24	22	36	30	R

The ribotyping of 28 strains and other reference strains showed that *Bacillus *species can be easily distinguished using this genotype characterization method. The ribotyping of 15 strains and calculation of the similarity values between the isolates and the reference strains allowed us to identify of all strains, which yielded a fingerprint identical to that of* B. cereus* reference strains 6066, 13209, and 6092. The ribotyping of 11 strains yielded a fingerprint identical to that of *B. pumilus *reference strains 6073, 11052, and 11055. The similarity of the fingerprint patterns allowed the grouping of the isolates with reference strains and their identification on this basis. 

PCA 24.1 and PCA 34.1 exhibited similar patterns to those provided by the *B. mojevensis* database (DUP-13240). Similarly, 11.2 showed patterns similar to those of the database for *B. subtilis* (DUP-18130). These findings show the high similarity among *B. pumilus *strains isolated from diabetic patients’ eyes.

Among the 28 isolates, 8 distinct *Eco*RI ribogroups were identified, and various resistance profiles were obtained. Table 1 summarizes the ribogroups found in this study. The antimicrobial susceptibility of the isolates was investigated using a panel of 10 different drugs, and the rates of resistant strains were as follows: methicillin 89.28%, gentamicin 3.57%, cefuroxime 71.43%, ceftazidime 71.43%, ciprofloxacin 3.57%, lomefloxacin 7.14%, and moxifloxacin 7.14%. No strain was resistant to amikacin, vancomycin, and gatifloxacin (Table 2). Among the ribogroups, the first group consisted of 14 isolates. The *B. cereus* KA 17.6-1 isolates belonging to the *Eco*RI-126-S-8 ribogroup were interestingly and strikingly related by their antibiotic resistance patterns, all being resistant to gentamicin, ciprofloxacin, lomefloxacin, and moxifloxacin.

*B. cereus* KA17.6.1 strains belonging to the *Eco*RI-126-S-8 ribogroups showed different antibiotic resistance patterns as compared to the other *B. cereus* strains. This strain was found to be resistant towards ciprofloxacin, gentamicin, lomefloxacin, and moxifloxacin. All of the *B. pumilus* ribogroups were resistant to cefuroxime, methicillin, and ceftazidime, while *B. subtilis *was resistant to moxifloxacin, and *B. mojevensis *PCA 34.1 was resistant to lomefloxacin.

Over 85.71% of the isolates were multiresistant to at least 2 drugs. Of these, 14.29% isolates were resistant to 2, 67.86% to 3, and 3.57% to 5 antibiotics. Only 1 strain of the 28 was susceptible to all the tested antibiotics.

## Discussion

With increasing numbers of diabetic patients worldwide, there has been a significant rise in the prevalence of diabetes-related eye problems. Few reports have examined the specific prevalence of *Bacillus* spp in this context. In contrast to previously published data, our study has shown the incidence of *Bacillus* spp in a significant number (30%) of diabetic eyes. *B. cereus, B. pumilus, B. mojevensis, *and* B. subtilis *were isolated*. *The predominant microbial flora was* B. cereus *followed by* B. pumilus. *Tekeli *et al* studied the flora of conjunctivas from diabetic patients but did not provide any information on *Bacillus *(25). Bilen *et al* isolated *Bacillus *spp from 8/132 (6.06%) diabetic patients and 1/50 (2.0%) non-diabetic patients (26). Coşkun et al. isolated *B. subtilis* (3.6%) from normal conjunctival flora (27). We isolated *B. subtilis* (3.57%) at similar frequencies in our study. Among the 28 isolates, 2 were identified to have *B. mojevensis*. This bacterium is not a pathogen and exhibits antimicrobial activity (28). The eyelids and conjunctival flora protect against pathogenic microbial colonization (29). Normal bacteriologic flora inhibits the growth and invasion of pathogenic bacteria by restricting their nutrition, limiting the space available for growth and secreting enzymes and antimicrobial substrates.

Notably, ribotyping with the RiboPrinter^®^ is an automated process that requires little preparation and has a rapid turnaround time. The reference DuPont identification database lists all the profiles of the reference strains for each species and is used for the comparative identification of each isolate. Eight ribogroups were identified by RiboPrinter^®^ analysis. 

The antimicrobial susceptibilities of the clinical isolates were investigated using a panel of 10 drugs. Almost all the strains were either resistant or multiresistant, particularly towards cefuroxime, methicillin, and ceftazidime. Methicillin resistance was probably because of the capacity of the bacteria to produce ß-lactamases. None of the strains was resistant to amikacin, vancomycin, and gatifloxacin. Other investigators have also reported similar antibiotic susceptibilities for clinical isolates of *Bacillus* spp (30-32). However, toxicity to retinal cells has been reported following amikacin use (33). Only six (21.43%) out of the 28 tested isolates were susceptible to ceftazidime. Previous studies have also reported a low prevalence of ceftazidime resistance among *Bacillus* spp (31). Twenty-seven (96.43%) out of the 28 tested isolates were sensitive to ciprofloxacin. In a similar study, the authors reported 97.1% inhibition of *B. cereus* by 1 µg/ml ciprofloxacin (34, 35). In another study, 15 out of 16* Bacillus* spp isolates expressed high-level ciprofloxacin sensitivity. *B. cereus *is often resistant to all ß-lactams, and serious infections are best treated with vancomycin or clindamycin, with or without an aminoglycoside. All the strains tested were inhibited by gentamicin except for *B. cereus* KA17.6.1. *Bacillus* spp was susceptible to aminoglycosides. Our results are similar to those obtained by Citron and Appleman (34).

Studies on the antibiotic resistance of *B. pumilus *and* B. mojevensis *are limited, because the organism is not pathogenic to humans or animals. However, some recent studies have revealed that several *Bacillus *species including *B. pumilus *can cause infections, ranging from skin infections to life-threatening bacteremia in immunocompromised individuals (18). Thus, more studies need to be performed to understand the human health significance of *B.* *pumilus*, genetic basis of infections, and resistance to antimicrobials (19, 36). 

## Conclusion

Microorganisms on conjunctival flora may represent the source of infection in certain situations such as ocular surgery, malnutrition, and immunocompromise. *Bacillus *spp cultured from ocular tissues or fluids should not be dismissed as contaminants.

However, we believe that in a high-risk population, species identification may be useful in diagnosing recurrent bacteremia, as evidenced by isolation of the same species, as opposed to contamination resulting from two species. Moreover, our findings of five *B. cereus* and three *B. pumilus* infections suggest that these species may be more pathogenic than other common species, such as *B. subtilis* or *B. megaterium*, in immunosuppressed hosts. 

We recommend culturing swabbed samples from diabetic patients to check for endophthalmitis. In culture-negative diabetic patients, the possibility of a *Bacillus* infection should be considered. Since a significant percentage of* Bacillus* isolates are penicillin resistant, we recommend that initial empiric antibiotic treatment of these infections consist of gatifloxacin, vancomycin, and amikacin.
